# Convergent differentiation of multiciliated cells

**DOI:** 10.1038/s41598-023-50077-5

**Published:** 2023-12-27

**Authors:** Shinhyeok Chae, Tae Joo Park, Taejoon Kwon

**Affiliations:** 1 Department of Biomedical Engineering, Ulsan National Institute of Science and Technology (UNIST), Ulsan, 44919 Republic of Korea; 2 Department of Biological Sciences, Ulsan National Institute of Science and Technology (UNIST), Ulsan, 44919 Republic of Korea; 3 Center for Genomic Integrity, Institute for Basic Science, Ulsan, 44919 Republic of Korea

**Keywords:** Ciliogenesis, Differentiation, Data integration

## Abstract

Multiciliated cells (MCCs) are epithelial cells that control body fluid flow and contribute to the clearance of pathogenic microbes and other particles from the airways, egg transport in oviducts, and circulation of cerebrospinal fluid in the central nervous system. Although MCCs have shared functions to control fluid flow via coordinated motility of multiple ciliary structures, they are found in multiple mammalian tissues originating from distinct germ layers and differentiate via distinct developmental pathways. To understand the similarities and differences of MCCs in multiple tissues, we investigated single-cell transcriptome data of nasal epithelial cells, bronchial tubes, fallopian tubes, and ependymal cells in the subventricular zone from humans and mice by cross-species data integration. Expression of cilia-associated genes was indistinguishable between these MCCs, although cell populations had unique properties by the species and tissue, demonstrating that they share the same final differentiation status for ciliary functions. We further analyzed the final differentiation step of MCCs from their distinctive progenitors and confirmed their convergent gene set expression for ciliogenesis at the final step. These results may provide new insight into understanding ciliogenesis during the developmental process.

## Introduction

Multiciliated cells (MCCs) are part of the mucociliary epithelium with multiple motile cilia primarily responsible for controlling the flow of biofluids on the surface^1,2^. In mammalian species, MCCs are mainly observed at three sites: the airway, oviduct, and ependyma. MCCs in the mucociliary epithelium of the airway propel mucus and protect the host by clearing pathogens and contaminants inhaled in the air^3^ and are targeted by pathogens that invade the host through the respiratory tract, including SARS-CoV-2^4,5^. Ependymal MCCs in the brain and spinal cord control the flow of cerebrospinal fluid and assist neuronal migration in ependymal tissues and ventricular development^6,7^. Similarly, MCCs in the oviduct epithelium generate flow on the surface that can efficiently pick up ovulated oocytes and move them toward the uterus^8^. Due to these multiple roles, genetic defects of motile cilia can cause congenital disorders collectively called motile ciliopathy^9^.

The molecular pathways that control the biogenesis and function of motile cilia are highly conserved across taxa, including unicellular ciliates like *Paramecium*, the Planarian flatworm, and most vertebrates^10^; therefore, MCCs in different species and tissues are anticipated to have similar molecular characteristics. However, MCCs in the three mammalian sites develop via distinctive developmental processes. Ependymal MCCs differentiate from radial glial cells (RGCs) in the central nervous system, which originate from the ectoderm along the neuronal cell lineages^11–13^. In addition to ependymal MCCs, other neuronal cells such as astrocytes, neuroblasts, and other glial cells also differentiate. On the other hand, airway MCCs differentiate from club cells along the lineages originating from the mesendoderm^14,15^, together with other cell types in the airway mucociliary epithelium like ionocytes and goblet cells, which are highly conserved across taxa^15–17^. MCCs in the epithelium of the female reproductive tract (FRT; oviduct in mice and fallopian tube in humans) differentiate from Müllerian ducts originating from the mesonephric coelomic epithelium^18^, together with secretory cells^19,20^.

Although the molecular characteristics of MCCs and biological pathways related to MCCs in different tissues have been studied in detail, the differences between these cells have not been extensively analyzed. Ivliev et al. examined the co-expression gene network based on microarray data of the brain, airway, and FRT^21^. More recently, Patir et al. performed a similar gene network analysis with GTEx data, which include extensive tissue gene expression^22^. Although these results provided novel cilia-related gene candidates because bulk tissue expression data with different cell types were mainly used, the contribution of MCCs in each tissue remains unclear. Furthermore, only human adult tissue data were used; therefore, it is unknown when MCC-specific gene expression patterns emerge and to what extent they are conserved between species.

Here, we investigated the molecular characteristics of MCCs in the ependyma, airway, and FRT, where they regulate body fluid flow, at the single-cell level. By integrating single-cell gene expression data of humans and mice, we analyzed the similarities and differences of MCCs from different tissues in these species based on expression of highly conserved orthologous genes. We found that MCCs from different species and tissues are distinctive, but display similar cilia-related gene expression. By analyzing developmental time-course data of MCCs in the ependyma and airway, we also confirmed that genes involved in ciliogenesis are turned on at a very late stage in these cells, in contrast with their progenitors (RGCs in the ependyma and club cells in the airway). Our results may help to understand the differences between MCCs in different tissues and at different stages, and the different phenotypes of patients with motile ciliopathy.

## Results

### MCCs from different tissues and species display distinctive expression patterns

**Figure 1:**
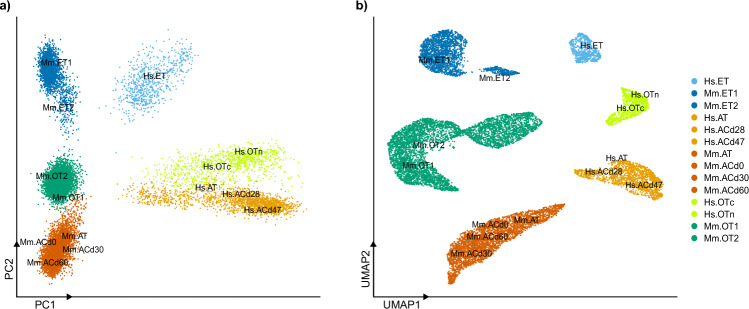
MCCs from different tissues have distinctive expression patterns. (**a**) PCA and (**b**) UMAP clusters show that MCCs from different tissues and species have distinctive gene expression patterns. However, independent datasets from the same tissue and species cluster together, confirming that there is no remarkable batch effect. According to the Elbow plot, PC1 and PC2 could explain 10.1% and 8.6% of variances, respectively.

To compare MCCs from different tissues and species, we merged 15,197 MCCs originating from 14 datasets into one matrix and performed cluster analysis. For cross-species comparison, we only considered 11,939 one-to-one matched orthologous genes between humans and mice, and performed additional normalization (see “Materials and methods” for details). With both PCA and the UMAP method, MCCs were separated into six clusters (Fig. 1). The first PC is the species difference and the second PC is the tissue difference. These results suggested that MCCs in different tissues and species have distinctive molecular characteristics, although they share cilia-related features. Similar to UMAP clustering, MCCs showed similar gene expression depending on the tissue and species in t-SNE clustering (Supplementary Fig. 3).

To investigate potential biological pathways underlying distinctions among species and tissues, we conducted a detailed analysis of differentially expressed genes (DEGs) and enriched biological pathways in each comparison. While our clustering analysis revealed the species difference (PC1) as the most prominent, we did not identify any enriched biological pathways among the DEGs when comparing humans and mice. This absence of enrichment may stem from inherent species differences despite the similar function of mature ciliated cells (MCCs) in both species. Nevertheless, we could not find any significant difference in biological pathways between fully developed MCCs in humans and mice. The second largest difference (PC2) corresponds to tissue variations, reflecting the developmental lineage of each MCC tissue, an aspect we further explored in subsequent analyses. The top 10 featured genes for each comparison (species-specific DEGs and tissue-specific DEGs) can be found in Supplementary Table 1.

We integrated relatively heterogeneous data; therefore, it is possible that these distinctive cell properties were due to a batch effect and other systematic biases rather than biological differences. To rule out this possibility, we checked the position of MCCs from each dataset labeled in Fig. 1. In both clustering methods, all cells from different experiments clustered together if they were derived from the same tissue and species, such as MCCs from human airway tissue (Hs.AT) and MCCs from in vitro culture-based differentiation experiments (Hs.ACd28). Therefore, we conclude that our results are unaffected by strong bias arising from the experimental conditions and reflect biological differences between MCCs from different tissues of humans and mice.

### Expression of genes related to ciliogenesis and ciliary functions is similar among MCCs

**Figure 2:**
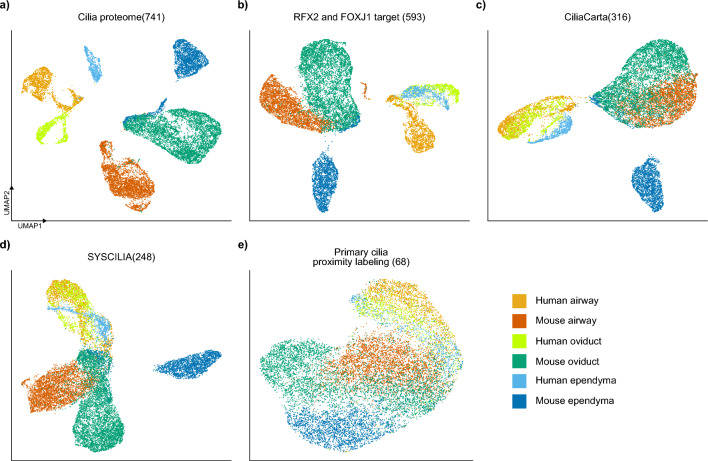
Differences among MCCs from different tissues are reduced when only cilia-associated genes are analyzed. MCC standard genes associated with ciliogenesis and ciliary functions were referred from the sources detailed below, and the UMAP was rerun. (**a**) Cilia proteome: the enriched ciliary structure proteome identified in the *Xenopus laevis* embryonic epidermis. (**b**) RFX and FOXJ1 target: genes regulated by the key transcription factors RFX2 and FOXJ1 for ciliogenesis. (**c**) CiliaCarta: a database of cilia-associated genes. (**d**) SYSCILIA: a manually curated list of cilia-associated genes. (**e**) Primary cilia proximity labeling: proteins identified in the ciliary structure, similar to the cilia proteome, but with proximity labeling of human cells. Although the species differences are retained, the difference among cell populations is reduced, indicating that common features associated with cilia are similar. Mouse ependymal cells were most distinguishable.

Although MCCs in each tissue are distinct from each other overall, they exhibit strong and similar gene expression patterns of cilia-related genes. Therefore, we wondered whether cilia-related genes are also differentially expressed in MCCs from different tissues. To investigate this, we analyzed five groups of cilia-related genes, namely, the ciliary axoneme proteome^23^, core MCC genes during ciliogenesis defined as targets of RFX2 and FOXJ1^24^, manually curated cilia genes from CiliaCarta^25^, SYSCILIA gold standard genes^26^, and the ciliary proteome determined by proximity labeling of primary cilia^27^ (Fig. 2). We only included one-to-one orthologous genes between humans and mice. For CiliaCarta data, we also filtered out genes with lower confidence (CiliaCarta score < 1).

Using these cilia-related gene matrices only, we performed UMAP analysis to determine differences among MCCs. Expression of the relatively large number of genes identified by high-throughput experiments differed among MCCs (Fig. 2a,b). By contrast, expression of the smaller number of refined cilia-related genes tended to be similar in different groups of MCCs (Fig. 2c,d).

MCCs from Mm.ET1 of mouse spinal cord ependyma showed a distinctive expression in this analysis (Fig. 2c,d). We found that genes related to developmental growth in CiliaCarta (GNAS, IFT80, DMD, MAGI2, and TTC8) and the immune response in SYSCILIA (PKD2, TRIM32, and MAL) are significantly different in Mm.ET1 compared to other MCCs. The original report of this dataset also mentioned that the enrichment of dividing microglia was probably due to dissociation-induced stress, which might affect this result even though we performed an additional normalization process^28^. However, other mouse ependymal MCCs from the brain (Mm.ET2) are well clustered with other MCCs. Also, when we only used the 68 genes identified in the human primary cilia axoneme by proximity labeling proteomics^27^, all groups of MCCs clustered (Fig. 2e). So we anticipate that the cilia-associated gene expression is still similar in MCCs of different tissues.

### Expression of genes related to motile ciliopathy does not differ among MCCs from different tissues

**Figure 3:**
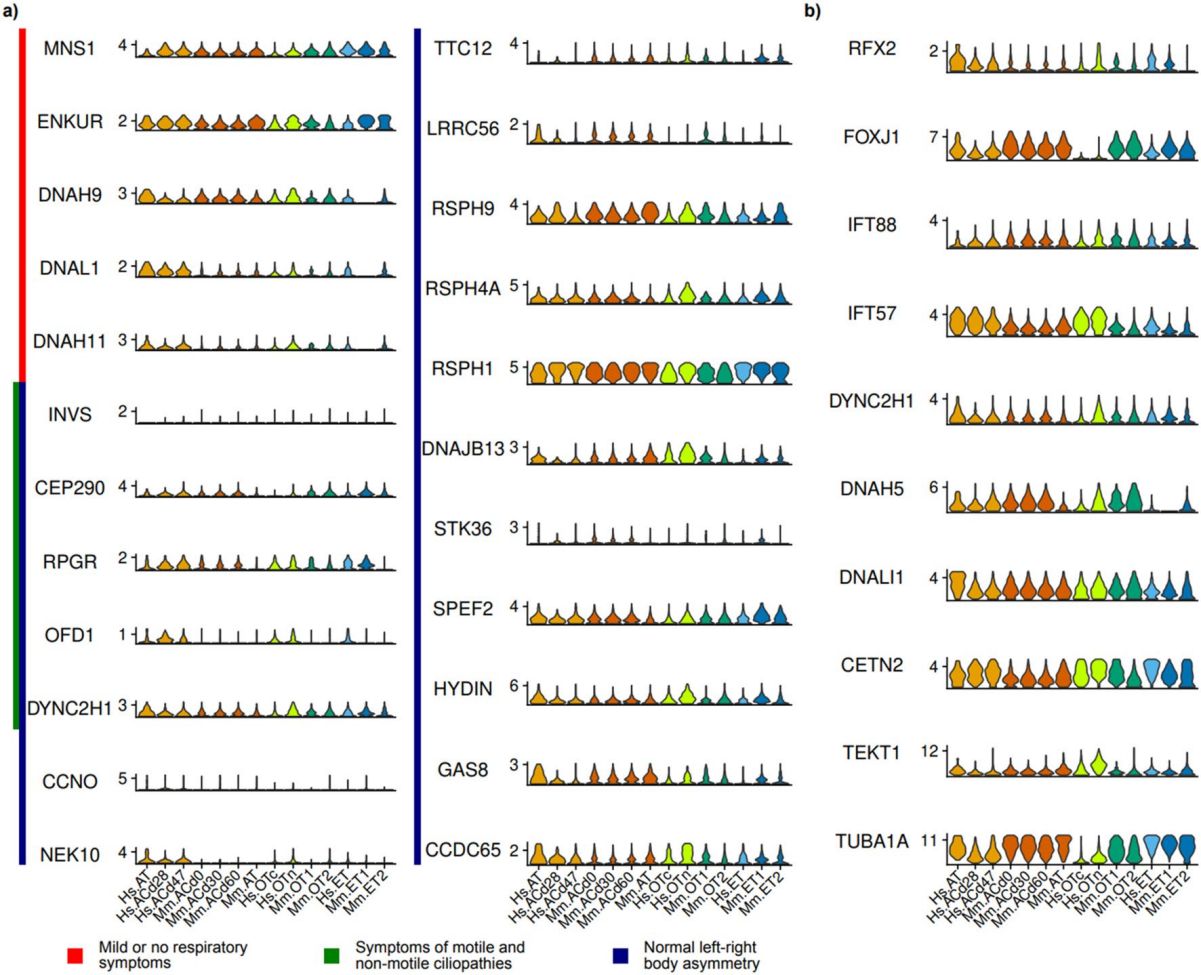
Gene expression patterns associated with ciliogenesis and ciliary function. (**a**) Violin plot of genes involved in ciliopathies according to the diagnostic outcomes of high-speed video microscopy and transmission electron microscopy analyses. Genes related to ciliopathies with mild or no respiratory symptoms, symptoms of motile and non-motile ciliopathies, and normal left–right body asymmetry were analyzed. Genes associated with symptoms of motile and non-motile ciliopathies and normal left–right body asymmetry include *RPGR*, *OFD1*, and *DYNC2H1*. (**b**) Violin plot of commonly used MCC markers. MCC markers include genes encoding transcription factors (*RFX2* and *FOXJ1*), intraflagellar transport proteins (*IFT88* and *IFT57*), the dynein complex (*DYNC2H1*, *DNAH5*, and *DNALI1*), centrin (*CETN2*), tektin (*TEKT1*), and tubulin (*TUBA1A*).

Motile ciliopathy is a genetic disease caused by dysfunction of motile cilia and causes symptoms in the tissues investigated in this study, such as respiratory system dysfunction (airway), central nervous system developmental disorders (ependyma), and fertility problems (FRT). Therefore, we investigated the tissue-specific expression of disease-associated genes^9^. If MCCs in different tissues have unique molecular characteristics, we anticipated that the expression patterns of genes related to motile ciliopathy will differ among MCCs in different tissues, which may be related to the physiologically diverse symptoms in different tissues (Fig. 3a).

We expected that ciliopathy-related genes whose mutations cause mild or no symptoms in the respiratory system might be expressed in other MCCs but not in airway MCCs. However, five such genes (*MNS1*, *ENKUR*, *DNAH9*, *DNAL1*, and *DNAH11*) were highly expressed in human airway MCCs. Expression of *DNAL1* and *DNAH11*, which encode subunits of dynein light and heavy chains, respectively, was relatively low only in mouse samples, although it did not significantly differ in airway MCCs. Expression of genes encoding all other members of the dynein complex did not show this distinction between humans and mice; therefore, we could not make any conclusion about the species specificity of the dynein complex. Further investigation is required to understand the differences in expression of these genes and their roles in ciliary functions, such as the roles of functionally redundant genes, which may differ between humans and mice.

The motile ciliopathies are also associated with heterotaxy, a developmental defect that causes left–right body symmetry^2,6^; therefore, we analyzed the tissue specificity of associated genes (Fig. 3a). However, similar to genes involved in respiratory system abnormalities, the expression patterns of these genes did not show any tissue specificity. Although the ciliopathy-related genes tested here were expert-curated and clinically relevant, they may not sufficiently reflect tissue-specific symptoms due to the clinical complexity of ciliopathy patients. Some genes, such as *OFD1* and *NEK10*, showed strong species-specific expression patterns. It may be interesting to compare their roles in ciliogenesis between humans and mice.

We also analyzed ciliopathy-associated genes in the Genomics England PanelApp^29^, annotated as "Rare multisystem ciliopathy super panel" (version 12.20). Among 86 genes listed in this panel, we only found one tissue-specific gene, namely, *TCTN3*, which was highly expressed in the FRT but not in other tissues. *DDX59*, *TMEM231*, *OFD1*, and *TCTN1* were expressed more in human tissues, while *ARL6* was expressed more in mouse tissues. However, most genes did not show tissue- or species-specific differences. Even though we could not identify tissue-specific ciliopathy-associated genes, it is still possible that we missed them. Because we only focused on the highly conserved genes between humans and mice, our analysis could not capture the contribution of species-specific genes. Furthermore, because we only analyzed the transcriptome of each cell population, we may have missed other alterations like post-transcriptional or post-translational regulation. Further studies with more direct samples related to the ciliopathy, such as patient samples or model animals, would be helpful to understand this tissue-specificity of ciliopathy.

In addition to ciliopathy-related genes, we analyzed expression of ten well-known cilia-related transcription factors and cilia structural protein genes (Fig. 3b). The expression levels of these genes and the percentages of expressing cells were very high without any tissue specificity. Although we found that *FOXJ1* expression was relatively low in human oviduct samples (Supplementary Fig. 1b), more than 50% of cells expressed *FOXJ1*. Expression levels of transcription factors are relatively low and tightly regulated over time; therefore, based on the number of cells, we think that *FOXJ1* is constantly expressed in all MCCs. Expression of *RFX2*, which encodes another transcription factor, showed relatively strong tissue-specific variation, although most MCCs exhibited distinctive expression of this gene compared with other cells. We concluded that known cilia-associated and ciliopathy-related genes are all commonly expressed in MCCs across three tissues of humans and mice.

### Tissue-specific DEGs in MCCs show the molecular signatures of progenitor cells

**Figure 4:**
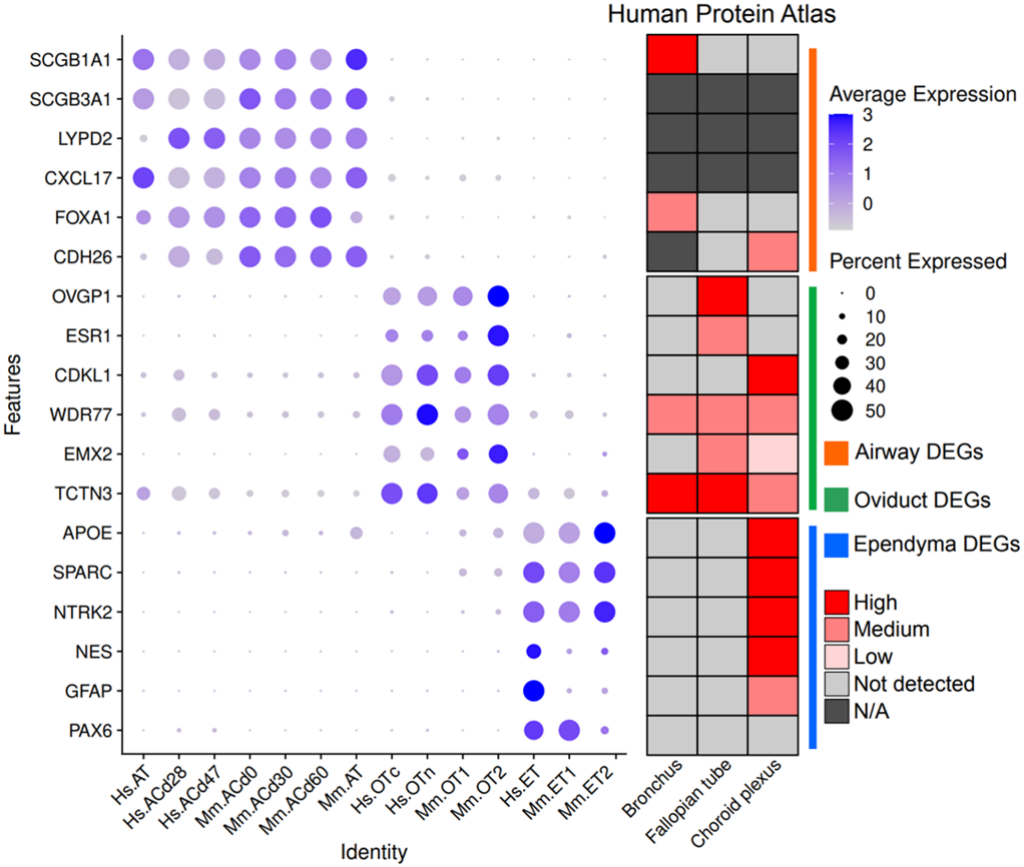
Tissue-specific DEGs. Dot plot of tissue specific DEGs. Genes with log2FC>1 in expression in each tissue compared with other tissues were chosen as DEGs. Expression heatmap shows the gene expression in the bronchus, fallopian tube, and choroid plexus from Human Protein Atlas normal tissue gene expression data.

To investigate which genes underlie the differences between MCCs from different tissues in both humans and mice, we analyzed DEGs with a greater than twofold change in mean normalized expression in MCCs from each dataset by comparing them with other cells in the population (Fig. 4). We excluded stochastically expressed genes (expressed in less than 10% of MCCs across tissues and species). To focus on differences between tissues, we filtered out genes with a greater than twofold change in mean normalized expression between species.

We found 40 airway-specific, 19 FRT-specific, and 266 ependyma-specific MCC DEGs. Airway MCC DEGs included epithelium development- and tissue homeostasis-related genes (GO:0001894) such as *PROM1* and *FOXC1*^30^. Moreover, *SCGB1A1* and *SCGB3A1*, which are well-characterized marker genes of progenitors of airway MCCs, were also expressed in airway MCCs^31^. FRT MCC DEGs included many reproductive process-related genes (GO:0022414) such as *ESR1*, *OVGP1*, and *GATA6*^32,33^. More than 100 ependymal MCC DEGs are involved in nervous system development according to GO analysis. Similar to airway MCC DEGs, ependymal MCC DEGs included marker genes of their progenitor cells (RGCs) such as *VIM*, *GFAP*, and *CLU*^6,12^. We confirmed genes with tissue-specific expression in a clustering plot (Supplementary Fig. 3). We concluded that each group of MCCs shows distinctive gene expression patterns due to their tissue-specific gene expression, which is associated with their developmental pathway. We also found 386 human-specific and 262 mouse-specific MCC DEGs but were unable to find any significant GO biological process (FDR < 0.05).

To validate the tissue-specific expression patterns of these genes, we also compared our results to the GTEx portal (version 8) and the Human Protein Atlas (version 23.0)^34^, using the closely related tissue data if exactly matched tissue is not available (lung and bronchus for the airway; spinal cord and choroid plexus for the ependyma). Even in this qualitative analysis, we confirmed that these tissue-specific DEGs of MCCs have similar tissue-specific expression patterns (Fig. 4). This result showed that MCCs in three different tissues share similar molecular characteristics not only to other MCCs but also to their neighboring cells in the same tissue. Even though we could not find any significant biological pathways enriched in each MCC, probably due to the high variations of integrated cell populations, these results could be useful resources to understand tissue-specific MCC differences. The full list of DEGs in each tissue MCCs is also provided in Supplementary Table 2 (GTEx) and 3 (Human Protein Atlas).

### Convergent differentiation route of MCCs in the airway and ependyma

Most of the differences between MCCs from different tissues were derived from the tissue identity; therefore, we reasoned that these MCCs differentiated distinctively but converged at a particular stage of development. Among the data analyzed, there were two developmental time-course datasets and therefore we could track the differentiation process. We identified ependymal MCCs and their progenitors (RGCs) in human fetal spinal cord data at week 11–25 of development^35^. We also identified MCCs and their progenitor club cells from human nasal epithelium culture data^14^. Club cells were present from day 7 after initiating nasal epithelial cell culture, but MCCs emerged from day 28. Therefore, we speculated that we could identify at which steps MCCs begin to establish their identity.

We used 14,524 RGCs and MCCs at weeks 11, 19, 24, and 25 of human fetal spinal cord development after removing batch effects and integrating them using CCA (Supplementary Fig. 4)^36^. After identifying those cells in their original UMAP based on the marker gene expression, we isolated them, including their neighboring cells, and performed the pseudotime analysis. Even though both RGCs and MCCs are available from weeks 11 to 25 of the human fetal spinal cord, we can clearly observe their differentiation lineages in the UMAP clustering results (Supplementary Fig. 4). Similarly, we analyzed 5799 airway MCCs and club cells (Supplementary Fig. 5).

**Figure 5:**
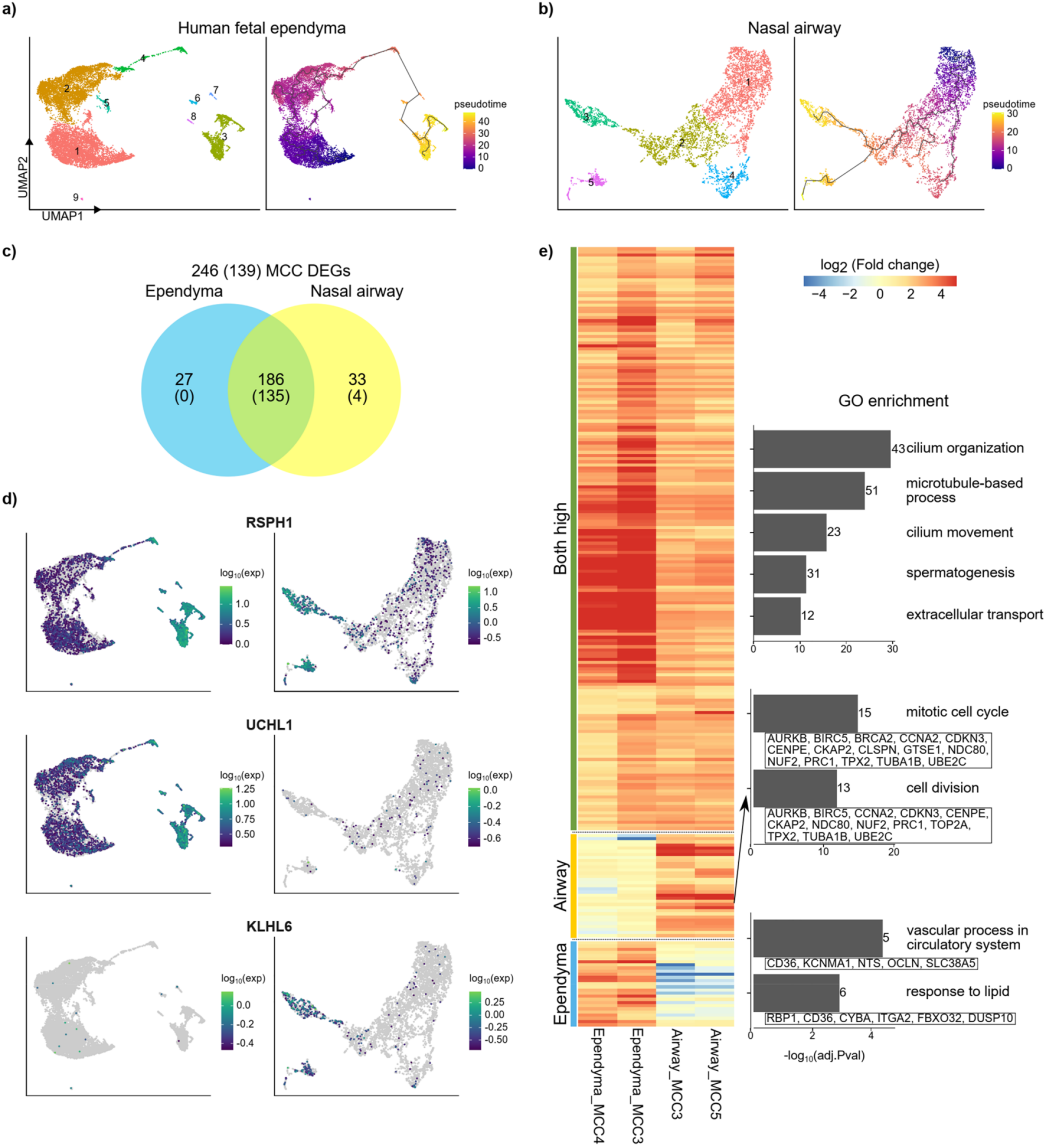
Pseudotime analysis of differentiation of MCCs in the airway and ependyma. Time-course gene expression data of human nasal airway culture and the human fetal spinal cord were analyzed. (**a**) Pseudotime clustering with time-series single-cell RNA-sequencing data of RGCs and MCCs from the human fetal spinal cord at 11, 19, 24, and 25 weeks. (**b**) Pseudotime clustering with time-series single-cell RNA-sequencing data of club cells and MCCs from the nasal epithelium (ALI culture data). (**c**) MCC DEGs compared with precursor cells among 608 overlapping variable genes selected for pseudotime clustering. Majority of them are also overlapped between MCCs from two tissues. (**d**) Heatmap and GO analysis of MCC DEGs (including upregulated MCC DEGs in both the airway and spinal cord, MCC DEGs in the airway, and MCC DEGs in the spinal cord) from pseudotime clustering. (**e**) Expression of MCC DEGs in each group.

Next, we performed pseudotime analysis with 2000 variable genes using monocle3^37^ to rearrange the isolated MCCs and their progenitor cells in ependyma and airway, according to the gene expression state and activity (Fig. 5). We also confirmed the transcriptome profile of known marker genes for MCCs and their progenitors through pseudotime (Supplementary Fig. 7). Based on this analysis, we identified 672 DEGs in ependyma (109 RGC upregulated genes and 563 MCC upregulated genes) and 881 DEGs in the airway (261 club cell upregulated genes and 620 MCC upregulated genes).

To compare MCCs with precursor cells, we first identified MCCs based on marker gene expression, the pseudotime graph, and the cell number in each cluster. We separated clusters #3 and #4 in the human spinal cord (Fig. 5a) and clusters #3 and #5 in the nasal airway dataset (Fig. 5b) as MCCs. Then, we compared the gene expression of the MCC clusters with that of the precursor cell cluster just before differentiation (cluster #2 for each dataset). We performed pseudotime analysis to identify the biological processes involved in differentiation based on these DEGs.

There were 608 overlapping variable genes between airway and spinal cord pseudotime analysis, 312 of which were MCC DEGs in at least one dataset (Fig. 5c). Of 312 MCC genes, 159 (51.0%) belonged to the five previously tested MCC databases (Fig. 2). To compare fold changes in gene expression against precursor cells from each dataset, we first removed genes with high fold changes that were very lowly expressed in each dataset (mean expression < 0.1). After filtering out these genes, we finally identified 246 MCC DEGs. Of these, 186 MCC DEGs were highly expressed in both datasets and 72.6% belonged to all five databases related to cilia-associated genes. This indicates that standard MCC genes are well conserved in both ependymal and airway MCCs. We also identified 27 and 33 DEGs expressed specifically in the spinal cord and nasal airway, respectively. For example, *RSPH1* was upregulated in both ependymal and airway MCCs, but *UCHL1* and *KLHL6* showed tissue-specific expression patterns (Fig. 5d).

To investigate concordance or discordance in the final step of MCC differentiation, we analyzed DEGs that were commonly upregulated in ependymal and nasal airway MCCs, which might be regulated uniquely in either cell type (Fig. 5e). In the case of commonly upregulated genes, cilia-related GO terms, such as cilium organization and microtubule-based process, were significantly enriched. Airway-specific MCC genes were mostly related to mitotic cell cycles and cell division, while ependyma-specific MCC genes were related to the response to lipid and the vascular process.

**Figure 6:**
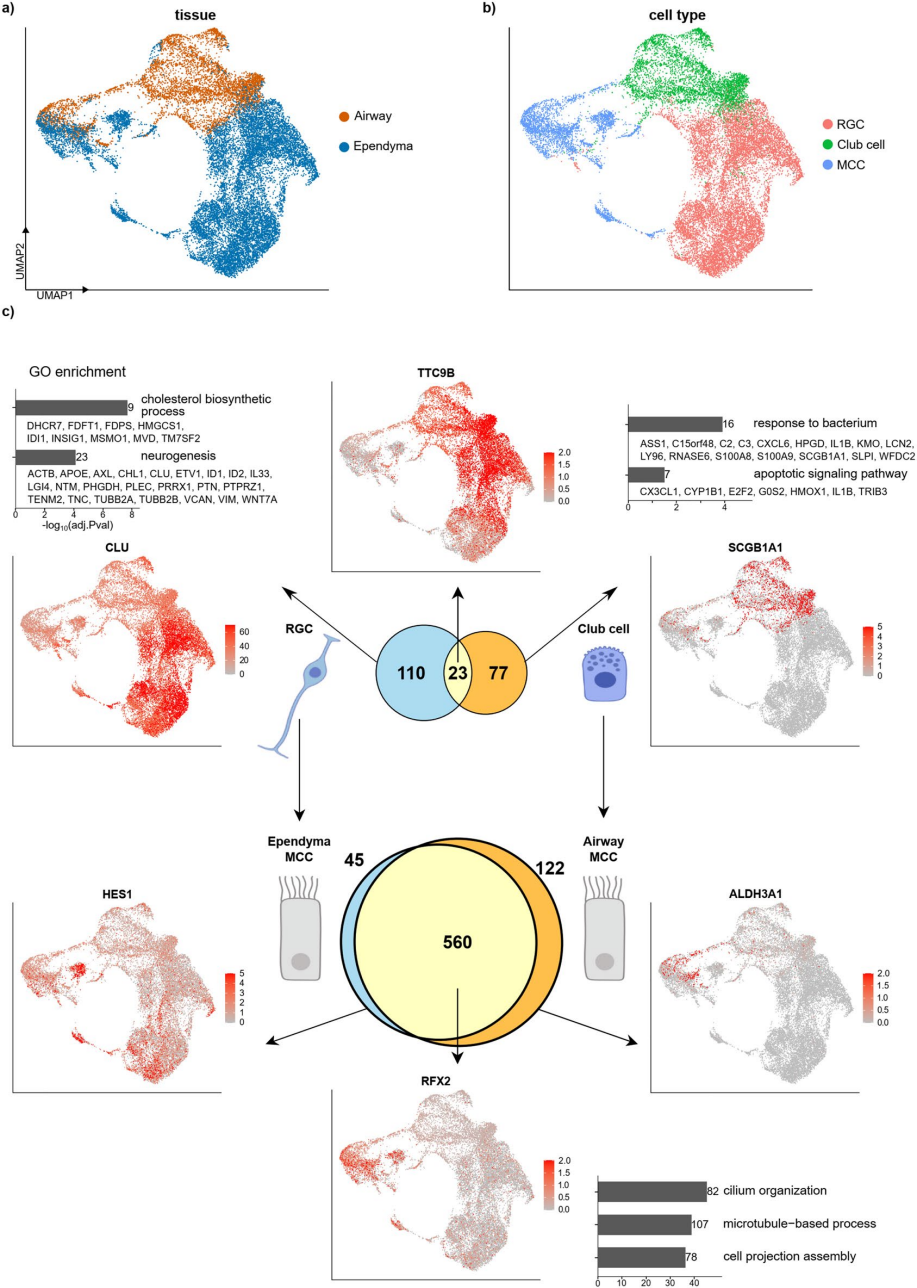
Cell integration analysis of MCCs and their precursor cells using human nasal airway culture and fetal spinal cord data. (**a**,**b**) UMAP clusters of integrated data show that MCC clusters merged, while precursor cell clusters were separate. (**c**) DEGs and their GO enrichment were analyzed using merged data. DEGs were first categorized as those in RGCs, in club cells, shared between precursor cells, in airway MCCs, in ependymal MCCs, and shared between MCCs. DEGs in RGCs, in club cells, and shared between MCCs were enriched with different GO terms.

To better understand these differentiation processes, we selected MCCs and their precursors from each tissue, integrated them by CCA using the 2000 most variable genes, and clustered them by UMAP (Fig. 6a,b). This clearly showed that RGCs and club cells were distinct. However, the differentiated MCCs clustered in both UMAP and t-SNE clustering, showing the convergent differentiation of MCCs from different progenitors (Supplementary Fig. 6). We identified 23 genes commonly expressed in RGCs and club cells, including *TTC9B*, but more genes showed precursor-specific expression (110 genes for RGCs and 77 genes for club cells) (Fig. 6c). The majority of MCC-specific genes overlapped between ependymal and airway MCCs. However, 45 and 122 genes were specifically expressed in ependymal and airway MCCs, respectively, such as *HES1* (ependymal MCCs) and *ALDH3A1* (airway MCCs). It might be interesting to study the tissue-specific functions of these genes in ependymal and airway MCCs. However, the majority of genes required to establish MCCs were convergently regulated upon differentiation from their progenitors in these tissues.

## Discussion

Recent advances in single-cell analysis allowed us to elucidate the molecular characteristics of various cell types across species, including a rare cell population we had not observed before. Following tremendous efforts to generate a catalog of these cell types in multiple species^38–41^, we can explore the similarities and differences among cells that share developmental pathways. This provides essential regulatory information to understand the cell differentiation process. By expanding this approach across multiple species, we began to explore the evolutionarily conserved molecular mechanisms that determine cell identity and functions^42^. However, compared with cells with similar functions in different species, cells with similar functions in the same species have not been intensively studied. Here, we showed that highly characterized MCCs in three mammalian tissues have distinctive gene expression patterns according to their developmental origins, although expression of most known genes associated with ciliogenesis and ciliary functions is conserved. All these tissues are extensively used to study ciliogenesis and motile ciliopathies, and we confirmed that they consistently express most known motile cilia-related genes. However, because their overall gene expression patterns are distinctive, it will be interesting to investigate the effect of these developmental backgrounds on ciliary functions.

Meta-analysis of single-cell data from different experiments is challenging due to the high sensitivity of this method. In addition to the computational preprocessing step, we utilized more than two independent datasets for each tissue, both from humans and mice, and confirmed that MCCs from the same tissue were more similar than MCCs from different tissues. It may have been possible to make this finding because the molecular characteristics of MCCs are very different from those of other cell types, and the same may not be true for all cell types. Indeed, in our recent study analyzing nine single-cell datasets from various mucociliary epithelium tissues, we found that MCCs have the most significant gene expression patterns compared with basal and secretory cell types^16^. By improving the experimental method to capture more cells and transcripts, this approach could become more helpful in understanding the conserved gene regulatory networks that establish the molecular characteristics of cells.

In addition to the molecular profiles of mature MCCs, we also analyzed their relationship with their progenitors in ependyma (human) and the airway (human and mouse). As expected, the gene expression signatures of RGCs (progenitors of ependymal MCCs) and club cells (progenitors of airway MCCs) did not overlap. Genes related to the cell cycle and cell division were differentially expressed in the airway. This may be because cells proliferate more in the airways than in the spinal cord because the airways are exposed to external substances. On the other hand, ependymal MCCs had a relatively small number of DEGs because the overall gene expression fold change compared with precursor cells was lower than in the airway. Nevertheless, expression of genes related to cell communication, such as *NTS*, *ITGA2*, and *UCHL1*, was specifically enriched in ependymal MCCs. We can use genes with tissue-specific expression to study the different genetic pathways underlying MCC development in each tissue. However, MCCs derived from RGCs and club cells clustered together; therefore, we propose that they "convergently differentiated", similar to convergent evolution. In contrast with the mammalian tissues investigated here, in which MCCs differentiate at very late stages, MCCs in the *Xenopus* mucociliary epithelium differentiate at an early stage and are maintained until maturation^16^. The datasets analyzed here are too sparse to cover all the steps of MCC differentiation and therefore we could not track these steps in detail. Further investigation of ciliogenesis in multiple tissues by performing high-resolution time-course experiments may help to understand the detailed regulation of differentiation from distinctive origin of early development.

Although our study showed that the MCCs of different tissues shared common expression patterns of cilia-associated genes, we are mostly focused on the fully differentiated MCCs based on well-characterized marker genes, so genetic effects that can alter the differentiation and other cell–cell communications might not be presented here. Further analysis of more detailed time course experiments and spatiotemporal data, together with the genetic variations, would help to decipher more details of the genetic effects on tissue-specific ciliogenesis, which can help our understanding of ciliopathy-associated phenotypes and the species difference between humans and mice.

Together with the tissue-specific differences, we observed differences between human and mouse samples. We initially speculated that this might help to explain the discordance between clinical ciliopathy symptoms and mouse models of ciliopathy. However, we could not find meaningful biological pathways to explain the difference between human and mouse MCCs. Our analysis may have been unable to capture species-specific ciliogenesis genes because it was limited to one-to-one pairwise orthologous genes. When we selected these orthologous genes from the original data, compared to the analysis with all genes in each species, the mouse data presented more different shapes than the human data. Therefore, if functionally redundant species-specific paralogous genes exist, our analysis might not have captured their roles. Detailed time-course experiments of each species and tissue that allow tracking of the cell differentiation process would also help to understand the differences between species.

## Materials and methods

### Data collection

We downloaded 14 single-cell transcriptome datasets from three sites of humans and mice with MCCs (airway, ependyma, and oviduct) from NCBI GEO^43^. Airway mucociliary epithelia data were from human bronchus tissue^44^, air–liquid interface (ALI) culture of human nasal epithelial cells^14^, and mouse tracheal epithelial primary cells^15^. Ependymal epithelia data were from the human fetal spinal cord^35^, mouse spinal cord^28^, and mouse subventricular zone^45^. In addition, we used two human fallopian tube datasets^46^ and two mouse oviduct datasets^19,47^. Detailed information, including accession numbers, is provided in Table 1.

**Table 1:** The single-cell transcriptome datasets analyzed in this study.

Label	Accession #	Species	Tissue	Total cell no	No. of MCCs	Description
Hs.AT	GSE131391	Human (N = 12)	Bronchial	814	84	Bronchial epithelium from smokers and non-smokers
Hs.ACd28	GSE121600	Human (N = 1)	Nasal epithelia	3557	300	ALI culture of human airway epithelial cells derived from nasal mucosa
Hs.ACd47	GSE121600	Human (N = 1)	Nasal epithelia	2775	1402	ALI culture of human airway epithelial cells derived from nasal mucosa
Mm.AT	GSE103354	Mouse (N = 6)	Bronchial	6152	409	Mouse tracheal epithelial cells
Mm.ACd0	GSE103354	Mouse (N = 3)	Bronchial	18,794	824	Differentiated mouse tracheal basal cells
Mm.ACd30	GSE103354	Mouse (N = 3)	Bronchial	24,229	996	Differentiated mouse tracheal basal cells
Mm.ACd60	GSE103354	Mouse (N = 3)	Bronchial	27,071	1511	Differentiated mouse tracheal basal cells
Hs.ET	GSE136719	Human (N = 2)	Spinal cord	39,952	750	Human fetal spinal cord data from aborted fetuses at week 24–25
Mm.ET1	GSE162610	Mouse (N = 3)	Spinal cord	12,488	2063	Uninjured spinal cord data from 8–10-week-old mice
Mm.ET2	GSE100320	Mouse (N = 5)	Brain	1700	299	Ependymal and neuronal cells from 8-week-old mice
Hs.OTc	GSE132149	Human (N = 5)	Oviduct	3877	465	Fallopian tube tissues from ovarian cancer patients
Hs.OTn	GSE139079	Human (N = 5)	Oviduct	1857	514	Fallopian tube tissues from ovarian benign patients
Mm.OT1	GSE180102	Mouse (N = 1)	Oviduct	515	286	Fallopian tube tissues from mice of reproductive age
Mm.OT2	GSE164291	Mouse (N = 16)	Oviduct	12,900	5294	Fallopian tube tissues from mice of reproductive age

### Individual data preprocessing

For cross-species analysis, we selected 11,939 one-to-one orthologous genes between humans and mice from Ensembl BioMart (Ensembl version 106)^48^. We used these genes even for data analysis of each species for consistency. We normalized each dataset based on these orthologous genes to have the same UMI count sum (3000) per cell and removed low-quality cells with fewer than 500 orthologous genes or multiplets with more than 6000 orthologous genes. We did not remove any further cells due to their low quality afterward. Then we followed the Seurat protocol (version 4.0.6)^49^ for further analysis. We selected 2000 variable genes with the "FindVariableFeatures" function, scaled them with the "ScaleData" function, and clustered cells by principal component analysis (PCA) or Uniform Manifold Approximation and Projection (UMAP) with the "RunPCA" or "RunUMAP" function, respectively. We determined the number of dimensions for analysis based on the "JackStrawPlot" and "ElbowPlot" results.

### Identification of MCCs

We used UMAP clusters to identify MCCs based on five cilia marker genes (*FOXJ1*, *RFX2*, *TEKT2*, *IFT57*, and *DYNLL1*)^10,50^. A cluster with strong marker gene expression was manually extracted as a new matrix. Then, MCC matrices from each dataset were merged using the "merge" function in Seurat for further analysis.

### Differentially expressed gene (DEG) analysis

We calculated expression fold change among MCC clusters using the normalized expression count output from Seurat. If the log2-transformed fold change (log2FC) of mean gene expression in each cluster was higher than 1 compared with the mean expression of cells in all other clusters and more than 10% of cells in the target cluster expressed the gene, we selected the gene as a marker of the target cluster. We confirmed DEGs by looking at violin plots and dot plots of expression in each dataset.

### Gene Ontology (GO) term enrichment analysis

We used marker genes identified by DEG analysis for pathway enrichment analysis via GO enrichment analysis with the PANTHER classification system (version 17.0)^51^ to determine tissue-specific gene functions. We analyzed genes with the "GO biological process complete" dataset in PANTHER. Then, we chose a significant pathway (FC > 2 and FDR < 0.05) with more than five genes in the list.

### Pseudotime trajectory analysis of MCC differentiation

We identified progenitor cells (RGCs for ependymal MCCs and club cells for airway MCCs) from the UMAP clusters based on marker gene expression (*APOE* for RGCs and *SCGB1A1* for club cells), and then isolated them in a separate matrix as performed for MCCs. To analyze gene expression changes during MCC differentiation in nasal epithelia and the spinal cord, we combined the MCCs and their progenitor cells separately and obtained pseudotime trajectory results from Monocle3 (version 1.0.0)^37^ with the 2000 most variable features. After UMAP analysis, we performed analysis with "JackStrawPlot" and "ElbowPlot" and identified 8 and 20 principal components (PCs), respectively.

### Cell integration analysis to remove the batch effect

Human fetal spinal cord data (accession number GSE136719) and comparison of MCCs with their precursor cells showed a strong batch effect; therefore, we performed canonical correlation analysis (CCA)^36^ in the Seurat package. To integrate samples with CCA, we first found a set of anchors with the "FindIntegrationAnchors" function using a default function. We then used the generated anchor sets to integrate data with the "IntegrateData" function. The same Seurat manual was used for integrated matrices.

### Supplementary Information

Supplementary Information.

## Data Availability

All data used in this study are available from the NCBI GEO. The detailed information, including accession numbers, is reported in Table 1.
