# Supplementary material for: Convergent differentiation of multiciliated cells
Source: Sci Rep. 2023 Dec 27;13:23028. doi: 10.1038/s41598-023-50077-5 (PMC10754865; doi:10.1038/s41598-023-50077-5)
Supplement: Supplementary file 1 — Supplementary Information. [file 41598_2023_50077_MOESM1_ESM.zip › MCCtissues.supplement.SciRep-rev1_1129.docx]

Supplementary Information

Convergent differentiation of multiciliated cells

Shinhyeok Chae^1^, Tae Joo Park^2,3*^, Taejoon Kwon^2,3*^

^1^Department of Biomedical Engineering, ^2^Department of Biological Science, Ulsan National Institute of Science and Technology (UNIST), Ulsan, 44919, Republic of Korea; ^3^Center for Genomic Integrity, Institute for Basic Science, Ulsan, 44919, Republic of Korea

# ^*^Corresponding Authors

**Taejoon Kwon**

Address: EB4(110) 701-8, UNIST-gil 50, UNIST, Ulsan, Republic of Korea, 44919

Email: [tkwon@unist.ac.kr](mailto:tkwon@unist.ac.kr); Phone: +82-52-217-2583

**Tae Joo Park**

Address: EB4(110) 701-4, UNIST-gil 50, UNIST, Ulsan, Republic of Korea, 44919

Email: [parktj@unist.ac.kr](mailto:parktj@unist.ac.kr); Phone: +82-52-217-2582

# Keywords: Multiciliated cells, Single-cell analysis, Ependyma, Female Reproductive Tract, Airway

# Supplementary Information

.

**MCCs are clearly identified in mouse and human tissues using common cilia marker genes.**

We collected the single-cell gene expression profiles of 156,681 cells after filtering out bad-quality cells from 14 single-cell transcriptome datasets from the airway, ependyma, and FRT of humans and mice. We first performed the standard preprocessing steps for each data matrix and clustered them with the UMAP method. We clearly identified MCC clusters that expressed well-known MCC markers (*FOXJ1*, *RFX2*, *TEKT2*, *IFT57*, and *DYNLL1*) (Supplementary Fig. 1a).

All datasets used were collected by different groups with various goals. Hence, the number of MCCs and their percentage in comparison with other cell types were highly variable between datasets depending on the experimental conditions (Table 1 in main text). It was essential to validate that the molecular features identified in this integrative analysis were representative of MCCs in each tissue of both humans and mice, without strong batch effects. Therefore, we collected at least two datasets for each tissue and investigated whether they clustered when combined (Supplementary Fig. 2). MCCs from different origins clustered separately, but those from the same origin mainly clustered together. More importantly, we could identify several hundred to more than 1,000 MCCs in each dataset, which were all distinguishable from other cells in the dataset.

To validate the MCCs identified, we further examined the expression levels of two cilia marker genes, namely, *DNALI1*, which encodes a putative member of the inner dynein arm orthologous to *Chlamydomonas* p28^23^, and *FOXJ1*, which encodes a transcription factor that regulates motile ciliogenesis^24^. In each dataset, we measured the expression of these genes in cells identified as MCCs and compared these expression levels with the average expression levels of the same genes in cells excluding MCCs (Supplementary Fig. 1b). Expression of *FOXJ1* in human oviduct tissue is relatively low. Hence, the difference in *FOXJ1* expression between MCCs and other cell types in this tissue was not reported (the p-value was still less than 0.001 based on the one-sided Student’s t-test). However, all other comparisons indicated that cilia marker gene expression was enriched in the MCCs identified but not in other cells. Based on this observation, we concluded that we collected a sufficient number of MCCs in each dataset for cross-species and cross-tissue comparisons.

# Supplementary Tables

**Supplementary Table 1.** List of top 100 featured genes (top 50 each for PC1 and PC2, respectively) in the principal component analysis in Fig 1.

**Supplementary Table 2.** Gene expression and reliability of tissue-specific MCC DEGs in Human Protein Atlas normal tissue data. 100 for High, 10 for Medium, 1 for low and 0 for Not detected or N/A. Bronchus, fallopian tube, and choroid plexus are selected to compare gene expression.

**Supplementary Table 3.** Gene expression of tissue-specific MCC DEGs in GTEx lung, fallopian tube, and spinal cord RNA-seq data.

# Supplementary Figures


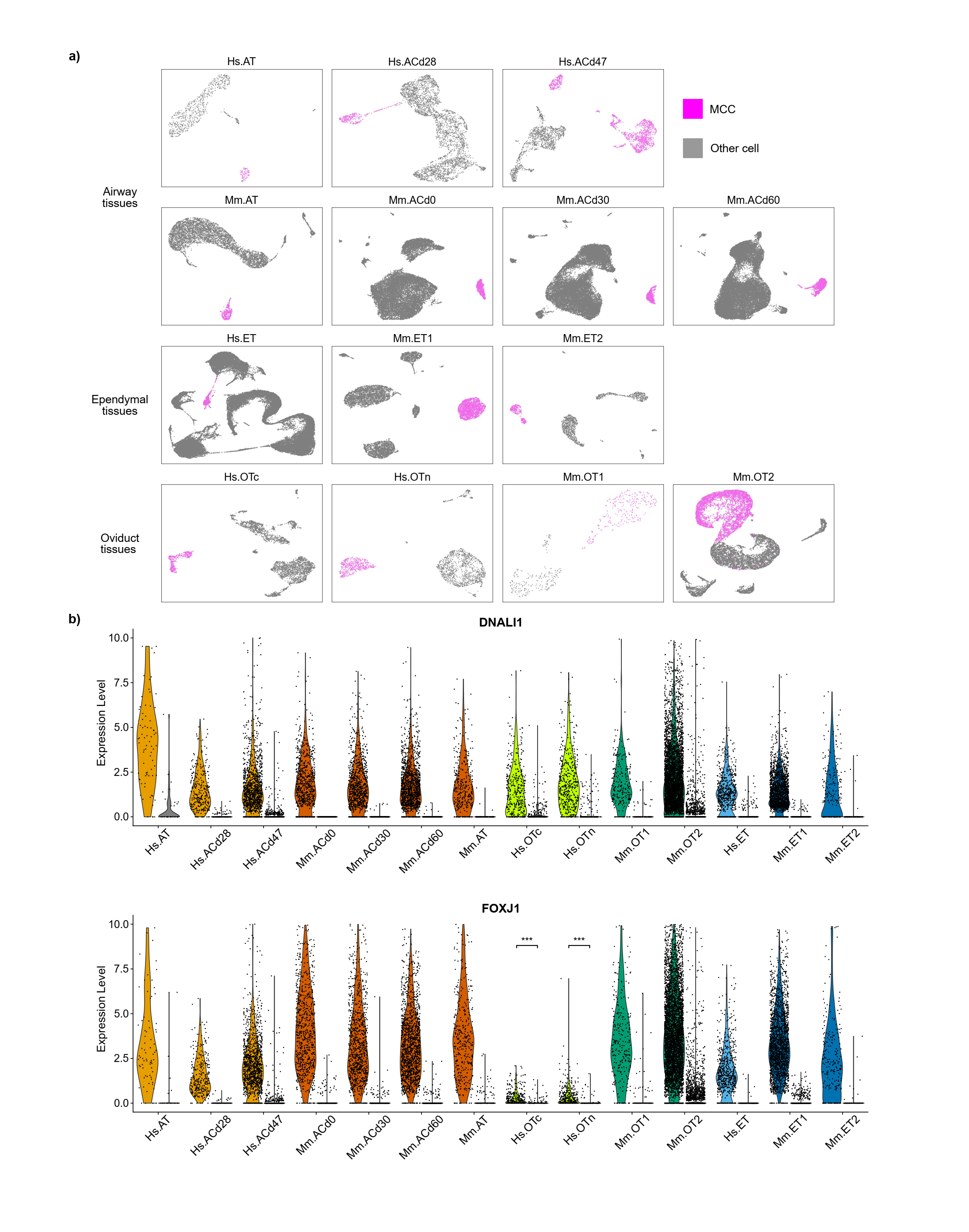


**Supplementary Figure 1. MCCs are identified in the airway, ependyma, and oviduct tissues.** **(a)** Transcriptome data from 156,681 cells were collected from various sources (Table 1) and clustered using UMAP to identify MCCs based on multiple cilia marker genes (listed in Supplementary Table 1). **(b)** Two representative cilia marker genes (*DNALI1* and *FOXJ1*) are highly expressed in the identified MCCs. Expression values in cells defined as MCCs are marked with colors, while those in other types of cells in gray on the right.

**
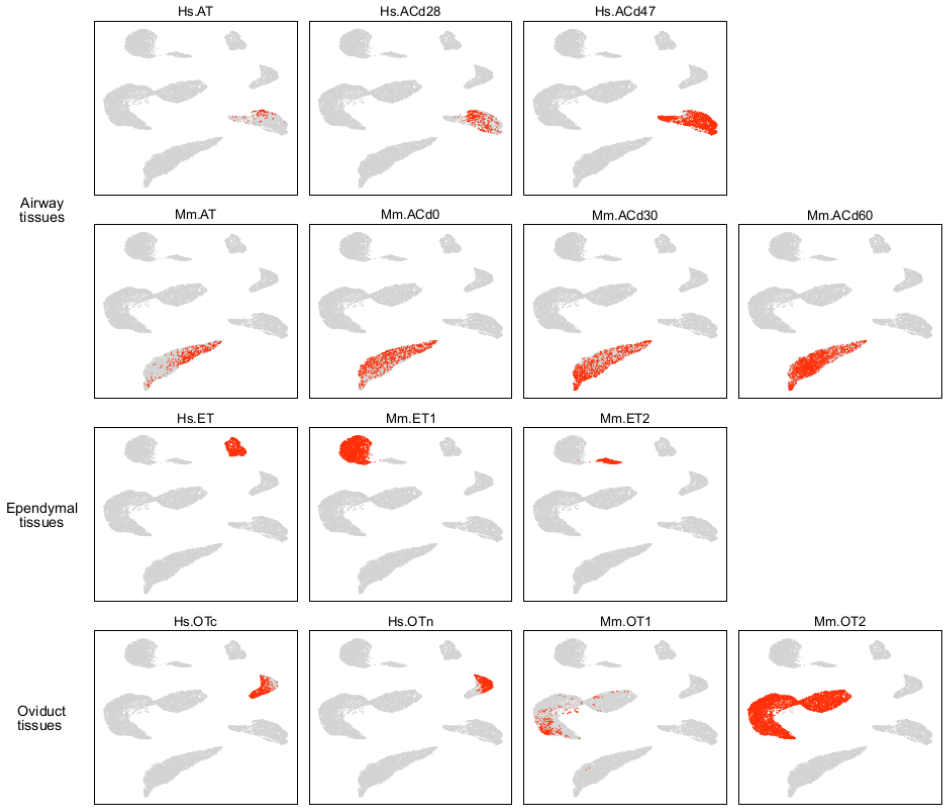
**

**Supplementary Figure 2.** **Distribution of MCCs from different tissues in UMAP clustering.** MCCs from each dataset were marked on UMAP clustering presented in Fig. 2.


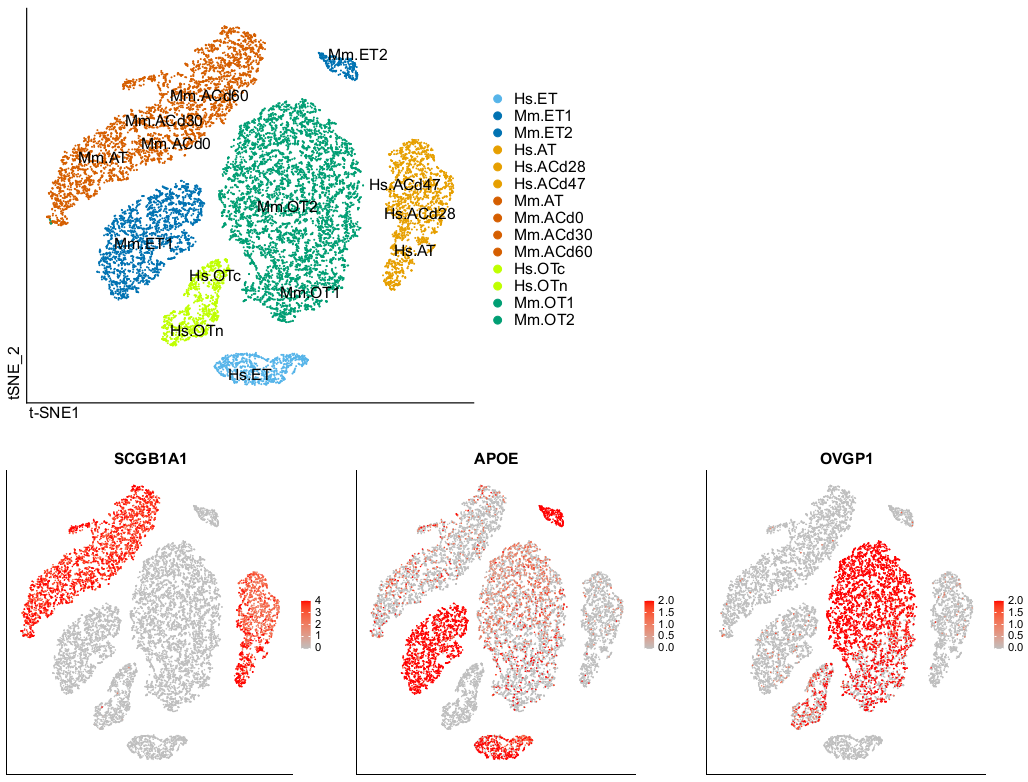


**Supplementary Figure 3. t-SNE clustering of MCCs from 14 datasets to test the robustness of clustering.** MCCs were clustered by t-SNE to test the robustness of clustering. MCCs from the same origin clustered together, while MCCs from mouse ependyma were separate. However, marker gene expression was similar depending on the tissue origin.


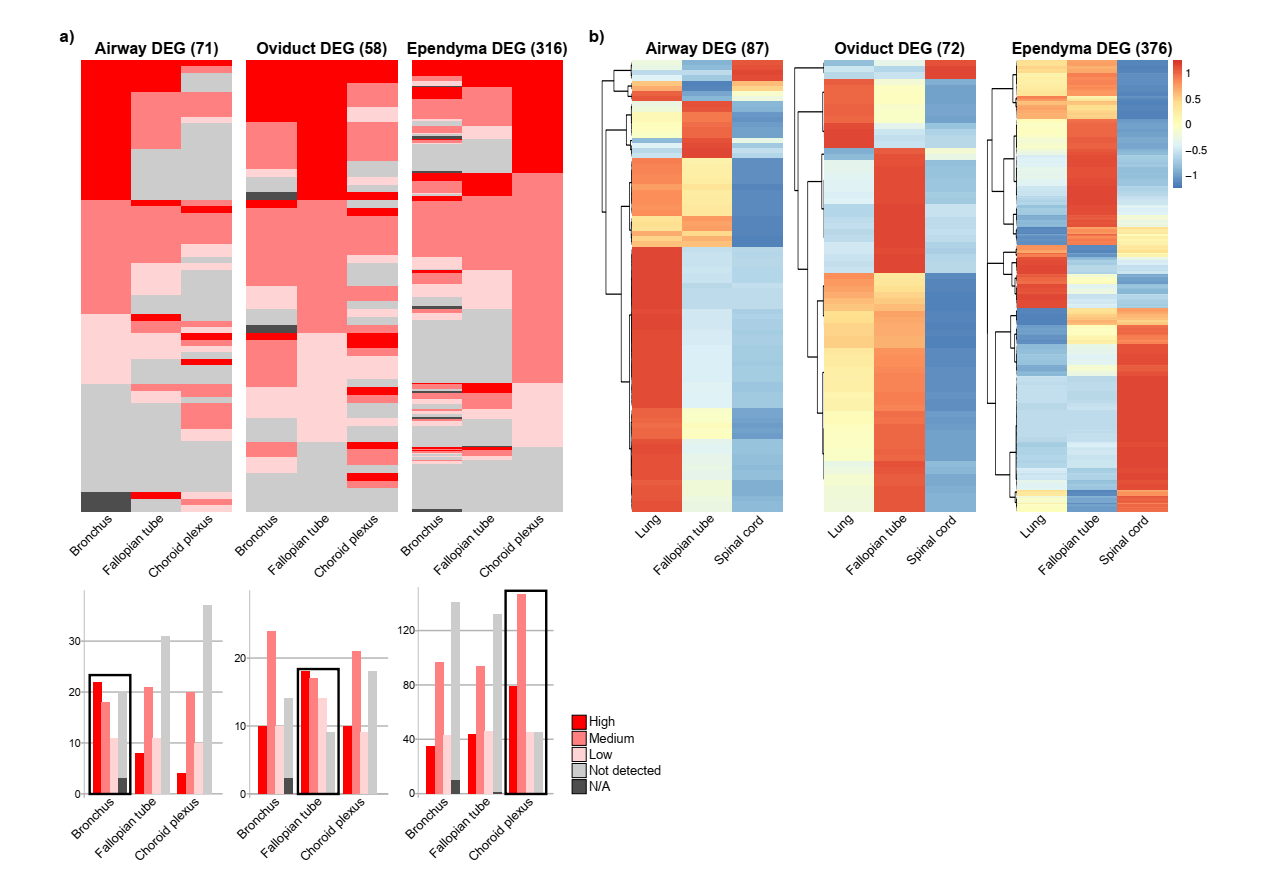


**Supplementary Figure 4. Comparison of tissue-specific MCC genes with gene expression data in bulk tissue from databases.** (a) Expression level heatmap and the number of each expression level of tissue-specific MCC genes from the Human Protein Atlas database. The bronchus, fallopian tube, and choroid plexus are selected to compare gene expression levels. The expression level is divided into high, medium, low, not detected, and no data in the Human Protein Atlas. (b) Expression heatmap of tissue-specific MCC genes from the GTEx portal (version 9) with Z-score. The lung, fallopian tube, and spinal cord are selected to compare gene expression levels.


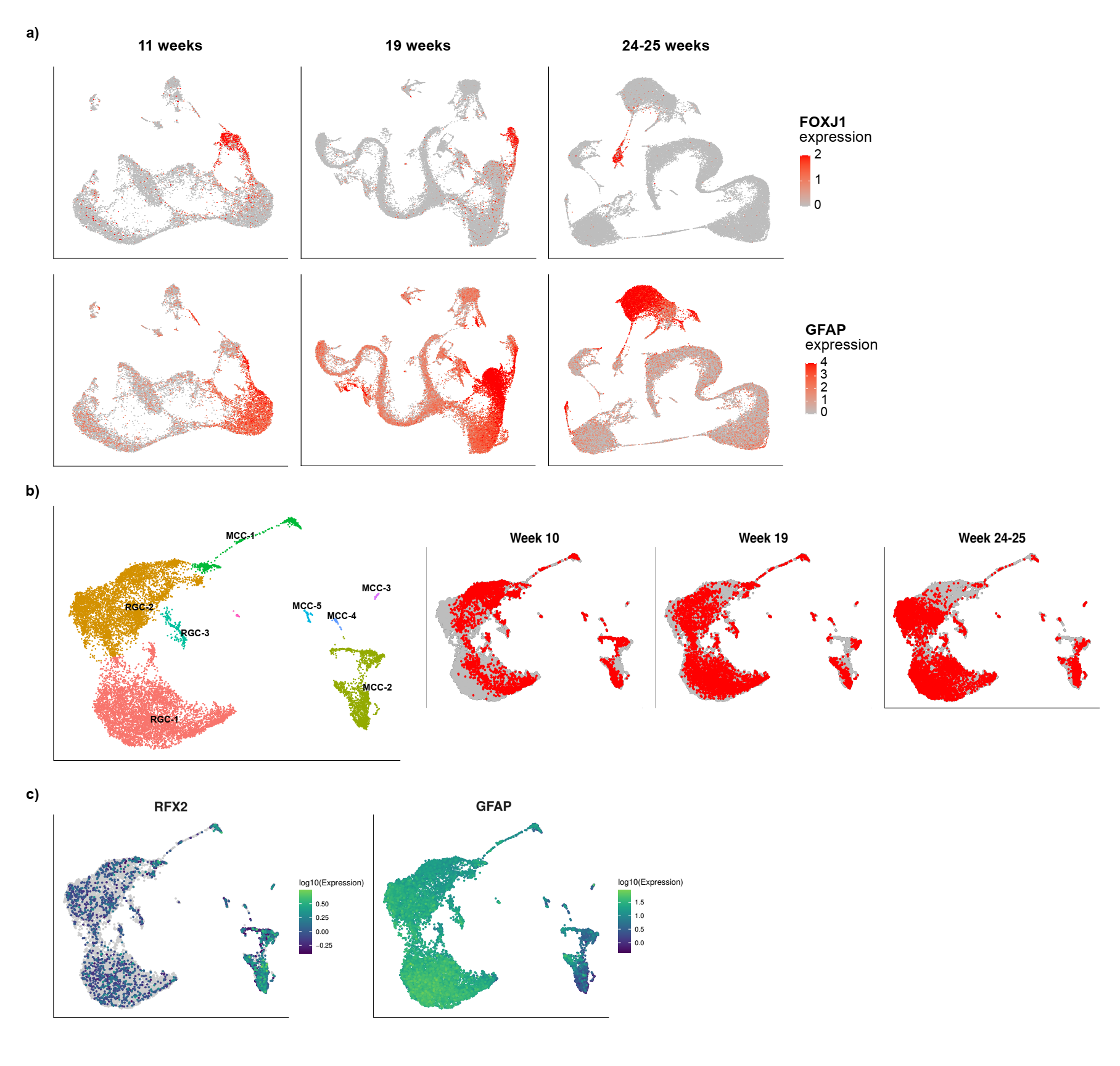


**Supplementary Figure 5. Marker gene expression to separate MCCs and RGCs.** Marker gene expression from human fetal spinal cord data was tested at various time points to separate MCCs and their precursor cells (RGCs). *FOXJ1* and *GFAP* were selected to define MCCs and RGCs, respectively. Cell clusters differentially expressing each marker gene were separated as MCCs and RGCs.


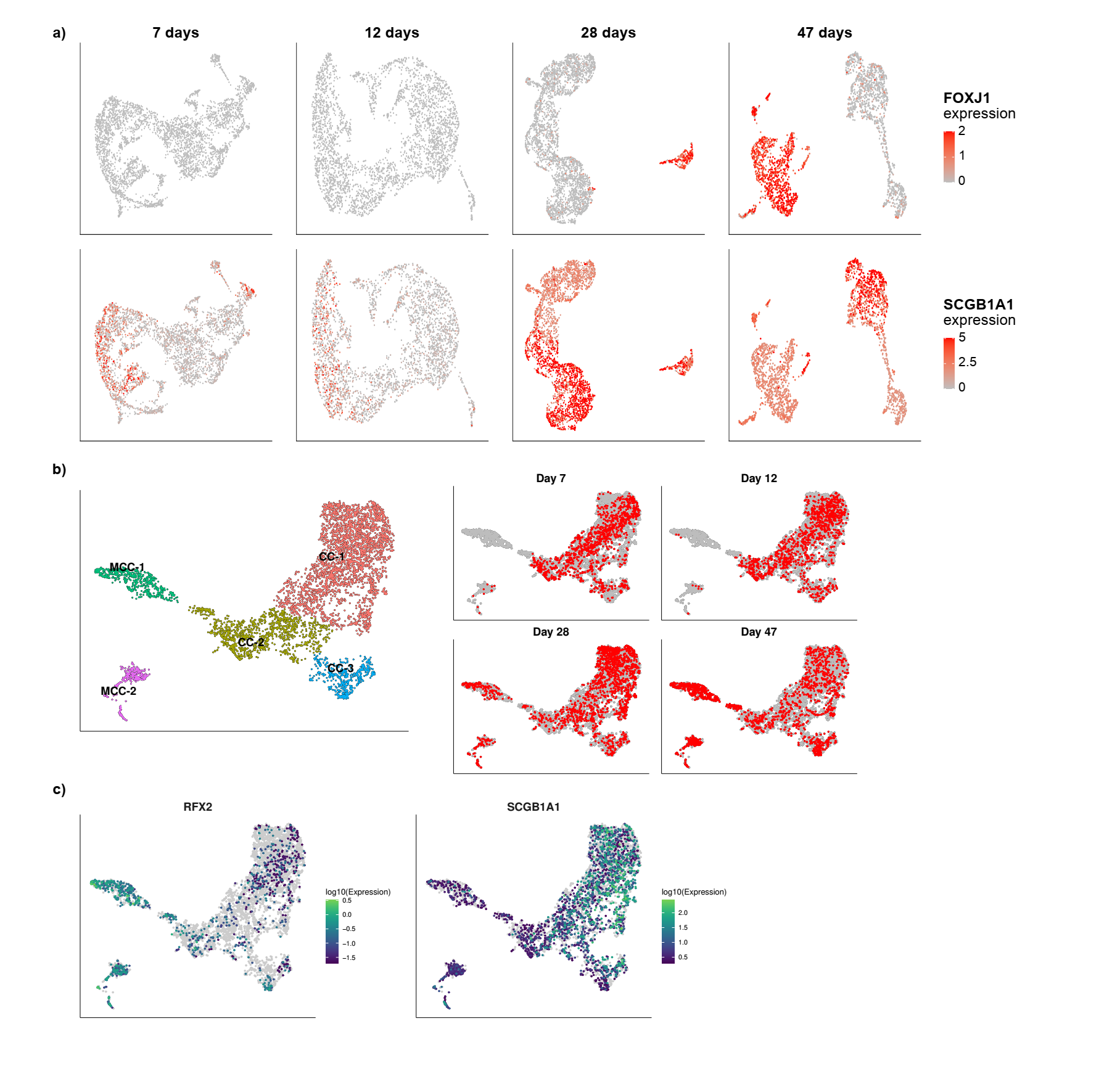


**Supplementary Figure 6. Marker gene expression to separate MCCs and club cells.** To separate MCCs and club cells in human nasal ALI culture data, expression of well-known marker genes of these two cell types was investigated. *FOXJ1* and *SCGB1A1* were selected to define MCCs and club cells, respectively. MCCs expressing both *FOXJ1* and *SCGB1A1* and club cells expressing only *SCGB1A1* at higher levels than MCCs were separated.


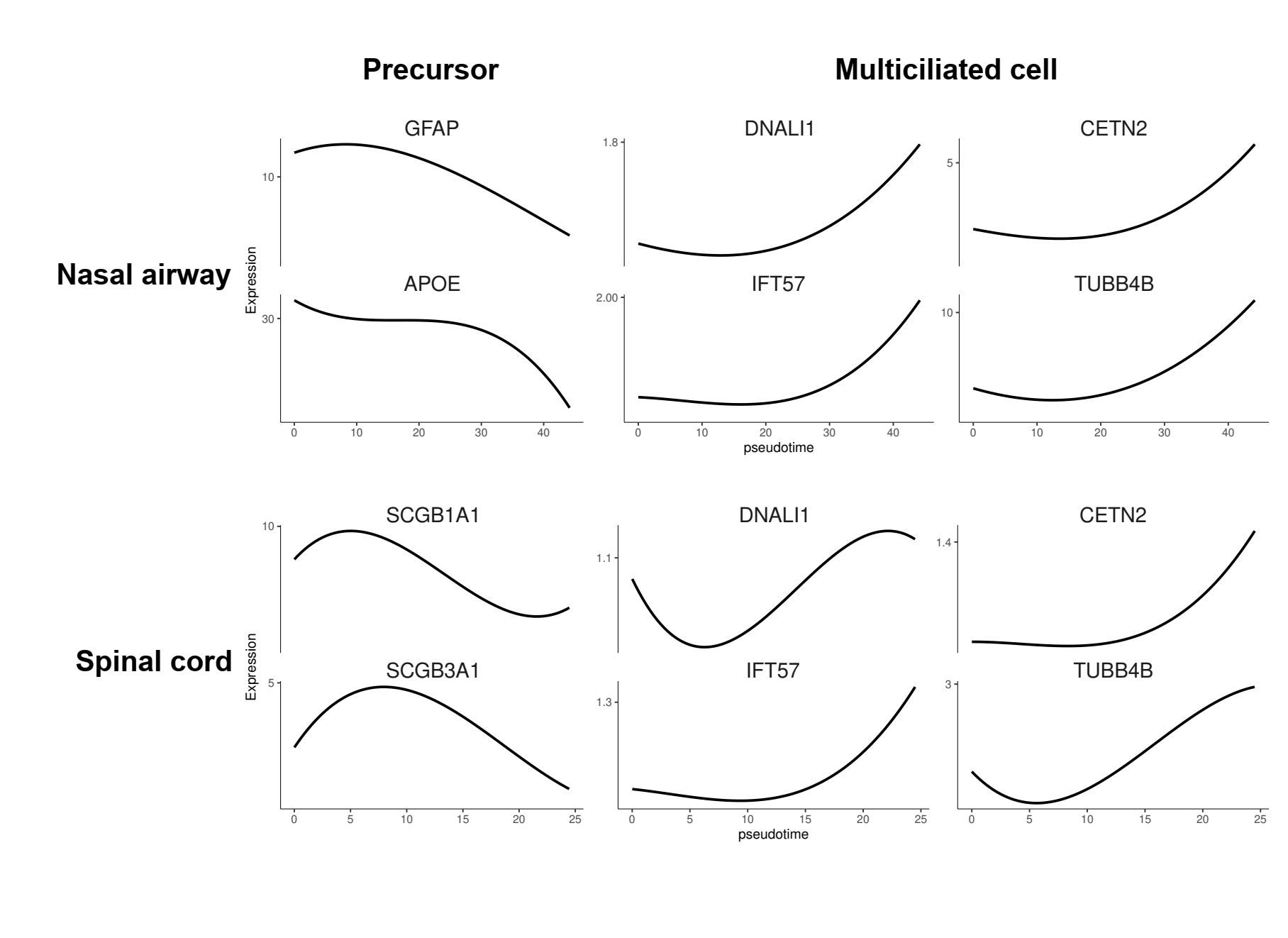


**Supplementary Figure 7. Expression patterns of marker genes for MCCs and their precursor cells according to the estimated pseudotime.** MCC marker genes gradually increase in both tissues as the precursor cell marker genes decrease. Due to many cells with empty expression values, we only presented the smoothed regression values of average expression for each pseudotime interval here.
